# Research progress on high-altitude hypoxia pulmonary injury: pathogenesis and Chinese herbal medicine for prevention and treatment

**DOI:** 10.3389/fphar.2026.1799029

**Published:** 2026-04-28

**Authors:** Jiale Song, Xiaojing Zhang, Hongfang Mu, Fuyixuan Zheng, Wenbin Li, Rong Wang

**Affiliations:** 1 Department of Clinical Pharmacy, The 940th Hospital of Joint Logistics Support Force of Chinese People’s Liberation Army, Lanzhou, China; 2 School of Pharmacy, Gansu University of Traditional Chinese Medicine, Lanzhou, China; 3 Department of Pharmacy, The 940th Hospital of Joint Logistics Support Force of Chinese People’s Liberation Army, Lanzhou, China

**Keywords:** Chinese herbal medicine, high-altitude hypoxia, high-altitude pulmonary hypertension, high-altitude pulmonary oedema, pathogenesis, prevention and treatment

## Abstract

Tourism in high-altitude areas and high-altitude outdoor activities have seen a significant increase, and health threats centered around high-altitude pulmonary edema and high-altitude pulmonary hypertension have become increasingly prominent. Hypoxia, as the primary pathogenic trigger, initiates a cascade linking inflammatory responses, oxidative stress, and vascular dysfunction, culminating in a vicious cycle of injury. Leveraging its unique advantages of holistic regulation and multi-targeted synergistic effects, Chinese Herbal Medicines (CHM) shows promising potential in preventing and treating pulmonary injury associated with high-altitude hypoxia. Through single herbs or compound formulations, CHM targets the HIF-1α pathway, scavenges reactive oxygen species, inhibits inflammatory cytokine release, protects the vascular endothelial barrier, and reverses abnormal vascular remodelling. This systematic review examines the core mechanisms by which high-altitude hypoxia causes acute and chronic pulmonary injury, and explores the action mechanisms of CHM in their prevention and treatment, aiming to provide a theoretical basis for the precise prevention and management of high-altitude hypoxia-related pulmonary diseases.

## 1 Introduction

The advancement of modern life and the demands of scientific research and military activities have led to an increasing number of people traveling to high-altitude areas. Based on the physiological responses exhibited by the human body in different high-altitude environment, elevations are categorised as follows low altitude (500–1,500 m), medium altitude (1,500–2,500 m), high altitude (2,500–4,500 m), very high altitude (4,500–5,500 m), and extremely high altitude (>5,500 m) (Peng, 2018). Medically, regions above 2,500 m in altitude are classified as high-altitude areas (Gong, 2022). High altitude exhibit a series of characteristics including low oxygen levels, low atmospheric pressure, cold temperatures, and intense ultraviolet radiation. Among these, hypoxia exerts a broad, non-specific impact on bodily functions and metabolism, constituting the most significant factor affecting human physiological processes in plateau environments (Li et al., 2019). The lungs serve as the body’s primary organ for gas exchange, whilst hypoxia constitutes the key factor inducing pulmonary tissue damage in high-altitude environment. CHM has a long history. It precisely intervenes in the key pathological mechanisms of lung injury caused by low oxygen levels in high-altitude environments, such as hypoxic adaptation disorder, inflammatory oxidative stress, vascular contraction and remodeling, and vascular leakage. In this article, we mainly summarize the specific pathogenesis of HAPE and HAPH, as well as the preventive and therapeutic effects of single Chinese herbs and Chinese herbal compound preparations on HAPE and HAPH in the hypoxic environment at high altitudes, as well as the target points. To provide a theoretical basis for further pharmacological research and clinical application of CHM in the prevention and treatment of high-altitude pulmonary injury diseases.

## 2 Epidemiology of pulmonary diseases induced by high-altitude hypoxia

High-altitude cough arises from persistent environmental irritation inducing bronchoconstriction, obstructing pulmonary secretion clearance and subsequently triggering coughing. A survey of military personnel stationed at high altitudes revealed a 32.96% incidence rate among individuals transitioning from lowland to plateau environments within 90 days, markedly exceeding the cough prevalence among university students in Guangzhou (10.9%) (Chen et al., 2006; Tang, 2024). Chronic Obstructive Pulmonary Disease (COPD) is characterised by airflow limitation that is partially reversible and progressively worsens, associated with abnormal pulmonary inflammatory responses to harmful gases or particulates (Halpin et al., 2021). High-altitude COPD is a chronic respiratory disorder defined by persistent airflow limitation, chronic airway inflammation, and progressive decline in pulmonary function. Its prevalence among individuals over 40 years old in China’s high-altitude regions stands at 9% (Chen Y. T. et al., 2023). High altitude pulmonary edema (HAPE) predominantly occurs in acute altitude sickness above 2,500 m. Its onset is sudden and rapid, causing significant bodily harm. As a severe non-cardiac pulmonary oedema, it is clinically diagnosed primarily by fatigue, dyspnoea, and dry cough during exertion (Gonzalez Garay et al., 2018), constituting the principal fatal cause of acute altitude sickness (Luks et al., 2019). Individuals with a prior history of HAPE exhibit a recurrence rate as high as 60%. Treatment mortality can reach 11%, while untreated mortality rates soar to 50% (Yang et al., 2019). High altitude pulmonary hypertension (HAPH) is caused by the special environment of high-altitude, low-pressure and low-oxygen conditions, which seriously threatens the life and health of both the settled and mobile populations at high altitudes. HAPH is also termed high-altitude cardiac disease or hypoxic pulmonary heart disease. The 2004 Sixth International Congress on High Altitude Medicine adopted the Consensus on Chronic and Subacute High Altitude Diseases, defining HAPH as high-altitude illness presenting with mean pulmonary artery pressure >30 mmHg or systolic pulmonary artery pressure >50 mmHg, accompanied by right ventricular hypertrophy, heart failure, moderate hypoxaemia, and without polycythaemia (León-Velarde et al., 2005). The prevalence of HAPH is approximately 6.2%, with higher rates observed in males than females and in the elderly compared to younger individuals (Gou et al., 2020). This paper therefore provides a systematic review of high-altitude pulmonary oedema and high-altitude pulmonary hypertension.

## 3 Pathogenesis of pulmonary injury in high-altitude hypoxia environment

### 3.1 HAPE

When the body rapidly ascends from lowland to high-altitude environment, hypoxia elevates pulmonary arterial pressure and increases pulmonary capillary circulatory resistance. This leads to heightened effective osmotic pressure within pulmonary capillaries, causing intravascular fluid to permeate into alveoli or pulmonary interstitium, thereby forming HAPE (Xu and Kong, 2020; Zheng et al., 2020). The pathogenesis of HAPE is primarily associated with hypoxia, inflammatory responses, oxidative stress injury, and vascular dysfunction.

#### 3.1.1 Hypoxia-induced oxidative stress damage

Inducible nitric oxide synthase (iNOS) catalyses the conversion of L-arginine into nitric oxide (NO) (Lee et al., 2019). As a key vasoactive molecule, NO plays a dual role in hypoxic adaptation. At low concentrations, NO dilates pulmonary vasculature, inhibits platelet aggregation, and mitigates hypoxic pulmonary vasoconstriction (Frostell et al., 1993). Conversely, at high concentrations, NO reacts with superoxide anion to form peroxynitrite (ONOO^−^), inducing lipid peroxidation and protein nitrosylation damage, thereby exacerbating vascular endothelial dysfunction (Kondrikov et al., 2014). In HAPE patients, excessive activation of iNOS disrupts NO production balance, causing substantial conversion of bioactive NO into ONOO^−^. This imbalance further exacerbates pulmonary vascular dysfunction and promotes pulmonary oedema formation (Beckman et al., 2000).

In pulmonary endothelial cells, the production of reactive oxygen species (ROS) originates from two sources. Under normoxic conditions, levels remain within the normal range, and it plays a key role in cellular signal regulation. A portion is derived from reduced nicotinamide adenine dinucleotide phosphate oxidase 2/4 (NOX2/4), which is expressed at low levels on the cell membrane and in the endomembrane system, generating trace amounts of ROS to regulate physiological processes such as cell proliferation, migration and vascular tone; Under hypoxic conditions, pulmonary neuroepithelial bodies (NEBs), acting as the core structures for hypoxia sensing, together with downstream axonal reflexes, constitute the local hypoxia sensing system within the lung (Hewitt and Lloyd, 2021). Hypoxia can activate NEBs and trigger axon reflexes, thereby mediating a disruption in non-adrenergic non-cholinergic (NANC) neuropeptide balance, which in turn upregulates NOX2/4 expression and induces massive ROS production, leading to and exacerbating oxidative stress damage Excessive ROS, in turn, activates the NEBs pathway and promotes the release of neuropeptides, thereby continuously exacerbating damage to the pulmonary vasculature and alveoli; this constitutes a key mechanism in the early initiation and progression of hypoxic lung injury (Kong et al., 2024). Another source is the mitochondria; as the cell’s ‘powerhouse’, their electron transport chain (ETC) is the primary source of ROS production. Complex III represents the core site for ROS production under hypoxia conditions (Wang et al., 2024). Under prolonged hypoxia conditions, dysfunction of the mitochondrial electron transport chain in endothelial cells occurs. During hypoxia, its structural stability is compromised, impeding electron transfer. This leads to leakage of electrons that combine with oxygen molecules, generating substantial ROS. These ROS are released from mitochondria into the cytoplasm of endothelial cells (Mao et al., 2024). ROS can inhibit the activity of endothelial nitric oxide synthase (eNOS). eNOS catalyses the conversion of L-arginine into NO, a key mediator for maintaining vascular homeostasis. NO exerts potent vasodilatory and anti-inflammatory effects, inhibiting inflammatory cell adhesion and thereby mitigating vascular endothelial injury (Berger et al., 2015; Shushanyan et al., 2025).

#### 3.1.2 Hypoxia-induced inflammatory response

The nuclear factor kappa B (NF-κB) pathway serves as a pivotal signalling pathway regulating inflammatory responses, with its activation status directly determining the expression levels of inflammatory mediators. Following acute high-altitude exposure, the ETC rate significantly slows, leading to substantial electron accumulation at complex III. This disrupts the coenzyme Q (CoQ) redox cycle, generating superoxide anion ·O_2_^−^ (Bell et al., 2007; Tian L. et al., 2023). The highly reductive·O_2_^−^ generated can be catalysed by superoxide dismutase (SOD) to form hydrogen peroxide (H_2_O_2_). H_2_O_2_ can diffuse through the mitochondrial membrane into the cytoplasm, where it acts as a signalling molecule to activate downstream inflammatory pathways (Nolfi-Donegan et al., 2020).

In the resting state, the NF-κB dimer p50/p65 heterodimer binds to the Inhibitor of Nuclear Factor-kappa B alpha (IκBα), remaining in an inactive form within the cytoplasm. IκBα binds to the p65 subunit via its ANK repeat domain, masking the Nuclear Localisation Signal (NLS) of NF-κB and thereby preventing its nuclear translocation, thus maintaining the quiescent state of the NF-κB pathway (Maimaitiaili et al., 2023). Hypoxia activates the inflammatory NF-κB pathway, whose activation promotes the release of inflammatory mediators such as tumour necrosis factor-α (TNF-α) and interleukin-6 (IL-6). These mediators directly damage vascular endothelial cells, disrupting tight junctions between endothelial cells and increasing vascular permeability. In animal models exposed to hypoxia, rat lung tissue exhibited significantly elevated levels of TNF-α and IL-6, positively correlated with increased vascular permeability (Shushanyan et al., 2025). ROS serve as key molecules in activating the NF-κB pathway, with their core mechanism involving oxidative modification of the Inhibitor of κB kinase (IKK) complex. The IKK complex comprises catalytic subunits IKKα and IKKβ, along with regulatory subunit IKKγ. IKKβ, as the core catalytic subunit, undergoes activity regulation that constitutes a critical step in NF-κB pathway activation (Hinz and Scheidereit, 2014). The ROS derivative ONOO^−^ oxidatively modifies the cysteine residues of IKKβ, releasing its self-inhibitory state and activating the IKK complex. Activated IKKβ possesses serine/threonine kinase activity, enabling specific phosphorylation of IκBα (Yee et al., 2021). Degradation of IκBα releases NF-κB dimers, which subsequently traverse the nuclear pore complex (NPC) to enter the nucleus, thereby facilitating nuclear translocation of the NF-κB complex (Da Silva-Ferrada et al., 2011; Popli et al., 2022). Concurrently, ONOO^−^ oxidatively modifies Endothelial Nitric Oxide Synthase (eNOS) structure, diminishing its activity and reducing NO production. This weakens NO’s vasodilatory effects and releases its inhibitory control over inflammatory responses (Hinton et al., 2023; Li et al., 2016), further amplifying NF-κB-mediated inflammatory activation (Hall et al., 2012; Huang, 2025). This establishes a vicious cycle: increased ROS production, reduced eNOS activity, and weakened inflammation suppression mutually reinforce ROS generation.

Upon activation, NF-κB primarily transcribes two categories of key inflammatory mediators (Cao et al., 2024). The first category comprises pro-inflammatory cytokines, including IL-6 and interleukin-8 (IL-8). As a multifunctional cytokine, IL-6 binds to IL-6 receptors on endothelial cell surfaces, activating the JAK/STAT3 pathway and further enhancing NF-κB activity (Hall et al., 2012). Forming an IL-6-NF-κB positive feedback loop. Studies indicate that after 72 h of hypoxia at 8,000 m altitude, IL-6 levels in alveolar lavage fluid and plasma were significantly elevated in the HAPE model group. Western blot analysis revealed markedly higher NF-κB content compared to controls, with a positive correlation between the two (Shi et al., 2021). Experimental studies indicate that TNF-α, IL-1β, IL-6, and IL-8 levels in lung tissue were higher in the model group than in the plainland group (Fang et al., 2025). As a neutrophil chemotactic factor, IL-8 attracts neutrophils from the vascular lumen to the pulmonary interstitium via concentration gradients. Its chemotactic activity depends on binding to CXCR1/CXCR2 receptors on the neutrophil surface (Teijeira et al., 2021). The second category comprises adhesion molecules such as Intercellular Adhesion Molecule 1 (ICAM-1) and Vascular Cell Adhesion Molecule 1 (VCAM-1), which serve as key structural mediators for the adhesion of inflammatory cells to endothelial cells (Pekarova et al., 2015), providing sites for inflammatory cells to traverse the vascular endothelial barrier (Pham et al., 2021). Adhesion molecule-mediated inflammatory cell adhesion constitutes a multi-step process, each stage dependent upon NF-κB-mediated expression of inflammatory mediators. Following transient binding and activation of inflammatory cells with endothelial cells, chemokines such as IL-8 and MCP-1 released by endothelium bind to corresponding surface receptors, thereby activating intracellular signalling pathways within inflammatory cells. This ultimately guides inflammatory cells through endothelial gaps to infiltrate the pulmonary interstitium (Shen et al., 2023). Macrophages and eosinophils that infiltrate the lung tissue can further amplify the hypoxia-induced inflammatory cascade, exacerbating damage to the endothelial barrier and vascular permeability. Upon activation by hypoxia and ROS, alveolar macrophages continuously secrete TNF-α, IL-6 and MCP-1 via the NF-κB and STAT3 pathways, forming a positive feedback loop of inflammation, whilst simultaneously releasing matrix metalloproteinases that directly degrade endothelial junctional proteins (Wang and Wang, 2023). Under the influence of chemokines, eosinophils are recruited and undergo degranulation, releasing major basic protein and eosinophil cationic protein, which directly damage the endothelial cell membrane and inhibit eNOS activity, thereby reducing NO production and synergistically promoting vasoconstriction and barrier disruption (Proper et al., 2014). The activation of macrophages can also amplify inflammatory signals via the NLRP3 inflammasome, recruiting further inflammatory leukocyte infiltration and creating a vicious cycle that significantly exacerbates the pathological progression of high-altitude pulmonary oedema (Zhou et al., 2024). This lays the groundwork for subsequent inflammation-cell-mediated damage to lung tissue.

#### 3.1.3 Hypoxia-induced vascular barrier damage

The occurrence of vascular barrier damage is not isolated but relies upon an upstream signalling network formed by hypoxia and inflammatory responses. Under normoxic conditions, prolyl hydroxylase domain-containing protein (PHD) catalyses hydroxylation modifications of proline residues Pro402 and Pro564 within the oxygen-dependent degradation domain (ODDD) of HIF-1α (Eltzschig et al., 2014; He et al., 2021). The PHD family comprises three subunits: PHD1, PHD2, and PHD3. Among these, PHD2—encoded by the EGLN1 gene—is the key subunit regulating HIF-1α stability (Berra et al., 2003; Sharma et al., 2022). Hypoxia directly inhibits the activity of PHD enzymes in the cytoplasm of pulmonary vascular endothelial cells (Richalet et al., 2023). Following reduced PHD activity, its hydroxylation modification function on HIF-1α is lost. Unhydroxylated HIF-1α cannot be recognised by the Von Hippel-Lindau protein (VHL), leading to its gradual accumulation and stable retention in the cytoplasm (Gojkovic et al., 2020; Yu et al., 2025). Stable HIF-1α undergoes nuclear translocation, entering the endothelial cell nucleus via nuclear pores. There, it binds to hypoxia response elements (HREs), initiating transcription of a cascade of downstream target genes (Frump et al., 2018). Vascular Endothelial Growth Factor (VEGF) represents one of the most critical downstream target genes of Hypoxia-Inducible Factor-1 alpha (HIF-1α). Upon binding to the HRE element within the VEGF gene promoter region, HIF-1α significantly enhances its transcriptional activity. This promotes increased synthesis of VEGF mRNA, which is then translated into protein and ultimately released into the extracellular space (Chen et al., 2025; Song et al., 2025; Sydykov et al., 2022). Concurrently, hypoxia disrupts the function of complex III in the mitochondrial electron transport chain of endothelial cells, generating substantial ROS. This activates the NF-κB pathway, leading to the release of pro-inflammatory factors (Wang et al., 2024). These inflammatory mediators act as upstream signals, synergising with VEGF to amplify vascular injury effects, with the core manifestation being an abnormal increase in vascular permeability. This key pathological basis of HAPE stems from the disruption of endothelial barrier integrity and the decline in alveolar fluid clearance function, involving functional abnormalities in multiple molecules such as tight junction proteins and aquaporins. VEGF, as the core downstream molecule of the HIF-1α pathway, is a key signal regulating vascular permeability. Extracellular VEGF specifically binds to the VEGFR2 receptor on the vascular endothelial cell membrane, activating the receptor’s tyrosine kinase domain and subsequently phosphorylating the downstream Src kinase. Acting as a signal transduction hub, Src kinase directly modulates VE-cadherin on the endothelial cell membrane. VE-cadherin is the core protein maintaining tight junctions between endothelial cells. Through its extracellular domain, it forms homodimers with VE-cadherin on adjacent cells, thereby sustaining barrier integrity (Sun et al., 2012). Src kinase-mediated tyrosine phosphorylation of VE-cadherin disrupts its complex with β- and α-catenin, leading to VE-cadherin dissociation from cell junctions and internalisation. This significantly widens intercellular gaps (Friedrich et al., 2019), permitting substantial leakage of proteins and fluid from the vascular lumen into the pulmonary interstitium. Consequently, the total protein concentration within the lung tissue increases.

Disruption of tight junctions mediated by claudin-5 and occludin further exacerbates impairment of endothelial barrier function (Garcia-Flores et al., 2022). Claudin-5 and occludin constitute core components of endothelial tight junctions, forming continuous barrier structures by binding to cytoplasmic junctional proteins such as ZO-1 and ZO-2, thereby preventing abnormal leakage of macromolecules (Jiao et al., 2021). Inflammatory cytokines can inhibit Claudin-5 and Occludin gene transcription and protein synthesis by activating the JAK/STAT3 pathway. Concurrently, hypoxia-induced ROS directly oxidatively modify the cysteine residues of Claudin-5 and Occludin, altering their spatial conformation and leading to disintegration of the tight junction structure (Shen et al., 2023; Villalba et al., 2023). Under hypoxic conditions, elevated IL-6 levels bind to IL-6 receptors on endothelial cell surfaces, activating the JAK/STAT3 signalling pathway (Huang et al., 2016) and directly suppressing the transcriptional expression of tight junction proteins Claudin and Occludin (Alsaffar et al., 2018). This represents one of the core mechanisms by which inflammatory mediators amplify abnormal vascular permeability.

#### 3.1.4 Hypoxia-induced changes in aquaporins

In addition to damage to the vascular endothelial barrier, reduced alveolar clearance capacity is another significant factor in the development of pulmonary oedema, a process closely associated with abnormal expression of aquaporins (AQPs) (Baloglu et al., 2020; Song et al., 2017). AQP5 is mainly expressed on the membranes of pulmonary alveolar type II epithelial cells, participating in the transport of fluid from the alveolar space into the interstitium (Flodby et al., 2017). Hypoxia and inflammatory responses synergistically suppress AQP1 and AQP5 expression (Fan, 2024). Hypoxia downregulates AQP1 expression by activating the HIF-1α pathway (Wittekindt and Dietl, 2019; Wu et al., 2023), while inflammatory mediators inhibit AQP5 expression via the NF-κB pathway (Towne et al., 2001). This results in reduced clearance efficiency of alveolar epithelial cells, leading to intracellular fluid accumulation.

### 3.2 HAPH

HAPH is a chronic pulmonary circulatory disorder characterised by pulmonary vascular remodelling and persistent elevation of pulmonary artery pressure, arising from prolonged exposure to hypoxia environment at high altitudes. It poses a severe threat to the health and lives of populations residing or working long-term at high elevations. Its core pathological features include pulmonary arteriolar muscularisation, vascular wall thickening, luminal stenosis, and progressive elevation of pulmonary artery pressure (Brito et al., 2020), ultimately leading to right-sided heart failure and even death (Sydykov et al., 2021).

#### 3.2.1 Hypoxia-induced vascular endothelial cell injury and proliferation

VEGF binds to VEGF receptors on pulmonary vascular endothelial cell membranes, promoting endothelial cell proliferation and migration while increasing vascular permeability. During the initial hypoxic phase of HAPH, its overexpression leads to abnormal proliferation of pulmonary arteriolar endothelial cells, compromising vascular endothelial integrity and laying the groundwork for subsequent vascular remodelling. During the initial hypoxic phase of HAPH, endothelin-1 (ET-1) expression levels correlate positively with pulmonary arterial pressure. Its overexpression induces sustained pulmonary vascular constriction, precipitating acute elevation of pulmonary arterial pressure. This process constitutes a key mechanism in the early development of pulmonary hypertension within HAPH (Yan et al., 2023).

#### 3.2.2 Hypoxia-induced proliferation and transformation of vascular smooth muscle cells

Pulmonary vascular smooth muscle cells are pivotal in regulating vascular tone and structural remodelling. Their hypercontractility and abnormal proliferation constitute the primary hallmark of vascular dysfunction in HAPH, directly leading to elevated pulmonary arterial pressure and vascular wall thickening. This process is driven by multiple signals including hypoxia and inflammatory mediators, achieved through the synergistic action of ETA/ETB receptor-mediated Ca^2+^ signalling pathways, ion channel regulation, transcription factor activation, and proliferative pathways. As the specific receptor for ET-1, the high expression and activation of ETA/ETB receptors on the smooth muscle cell membrane are pivotal for enhanced vasoconstriction. Under prolonged hypoxic conditions, pulmonary vascular endothelial cells secrete substantial amounts of ET-1 (Berger et al., 2009). This factor binds with high affinity to ETA receptors on the smooth muscle cell membrane, rapidly activating phospholipase C (PLC) in the cytoplasm. Upon activation, PLC catalyses the hydrolysis of phosphatidylinositol 4,5-bisphosphate (PIP_2_), yielding inositol trisphosphate (IP_3_) and diacylglycerol (DAG). IP_3_ specifically binds to IP_3_ receptors on the endoplasmic reticulum (ER) membrane, triggering the rapid release of Ca^2+^ stored within the ER into the cytoplasm. This process causes a transient, significant increase in cytoplasmic Ca^2+^ concentration. Ca^2+^ binds to calmodulin (CaM) to form a complex, activating myosin light chain kinase (MLCK). This promotes phosphorylation of myosin light chains, ultimately inducing smooth muscle cell contraction (Zhao et al., 2021). Concurrently, DAG activates protein kinase C (PKC), which further phosphorylates downstream contraction-related proteins, enhancing smooth muscle cell contractile force. Abnormal ion channel function represents another crucial mechanism underpinning enhanced smooth muscle cell contraction and proliferative activation, with the synergistic regulation of potassium voltage-gated channels (KV channels) and voltage-gated calcium channels playing a central role. Hypoxia directly inhibits the activity of KV channels on the smooth muscle cell membrane, reducing potassium efflux and causing depolarisation of the intracellular membrane potential (Wu et al., 2017). Membrane depolarisation activates voltage-gated Ca^2+^ channels, promoting substantial Ca^2+^ influx and further elevating cytoplasmic Ca^2+^ concentration. This synergises with IP_3_-mediated Ca^2+^ release from the endoplasmic reticulum, persistently enhancing smooth muscle cell contraction (Oka et al., 2008). Notably, Ca^2+^ influx not only regulates contractile function but also activates downstream mitogen-activated protein kinase pathways. Upon ERK1/2 activation, nuclear translocation occurs, phosphorylating cell cycle-related transcription factors. Concurrent activation of Rho kinase and CaM further amplifies the synergistic effects of contraction and proliferation in smooth muscle cells (Moral-Sanz et al., 2016). Activation of ETA receptors initiates not only the PLC-Ca^2+^ signalling pathway but also activates the Rho/Rho kinase pathway via G protein-coupled receptors. Activated Rho kinase phosphorylates the regulatory subunit of myosin light chain phosphatase, inhibiting its activity and reducing myosin light chain dephosphorylation. This enhances smooth muscle cell contractile tension and maintains the contracted state (Kovacs-Kasa et al., 2016). Concurrently, CaM, upon Ca^2+^ binding, not only activates MLCK but also initiates CaM-dependent phosphorylation of ERK1/2 and Akt, synergistically promoting Cyclin D1 expression and cell cycle progression via the mTOR pathway (Roskoski, 2012). As a central integrator of proliferative signals, mTOR activation directly regulates ribosomal protein S6 kinase and translation initiation factor 4E-binding protein 1, enhancing Cyclin D1 translation efficiency and accelerating smooth muscle cell proliferation. HIF-1α, acting as a transcription factor, plays a pivotal regulatory role in smooth muscle cell proliferation and angiogenesis. Prolonged hypoxia directly activates HIF-1α within smooth muscle cells, enabling its nuclear translocation to bind promoter regions of proliferation-related genes such as VEGF and platelet-derived growth factor (PDGF), thereby promoting their transcription and expression (Ishizuka et al., 2011). Prolonged hypoxia can lead to the sustained release of calcitonin gene-related peptide (CGRP), 5-hydroxytryptamine (5-HT) and ET-1 from pulmonary NEBs, thereby causing chronic pulmonary vasoconstriction and a persistent increase in vascular resistance (Hanthazi et al., 2019). Long-term abnormal elevations in ET-1, CGRP and 5-HT can further induce endothelial dysfunction, abnormal smooth muscle proliferation and fibroblast activation, ultimately driving pulmonary vascular remodelling and progressing to persistent pulmonary arterial hypertension (Keegan et al., 2001; Yan et al., 2024). Following release into the extracellular space, VEGF and PDGF bind to corresponding receptors on the smooth muscle cell membrane via autocrine and paracrine mechanisms. This activates the PI3K/Akt/mTOR pathway, amplifying proliferative signals while simultaneously promoting angiogenesis. This leads to thickening of the smooth muscle layer in pulmonary arterioles and abnormal proliferation of the vascular network, exacerbating luminal narrowing (Zhao et al., 2021). Imbalance between NO and ET-1 constitutes a key hallmark of vascular dysregulation in HAPH. Mirroring the pathogenesis of high-altitude pulmonary oedema, VE-cadherin—a critical component of intercellular junctions in endothelial cells—dissociates from adhesion sites, enters the cytoplasm, and undergoes degradation. This disrupts endothelial cell adhesion, enlarging intercellular gaps. This process markedly increases vascular permeability, allowing plasma proteins and fluid to leak from the vasculature into the pulmonary interstitium. Concurrently, it provides pathways for inflammatory cell infiltration, exacerbating the formation of a local inflammatory microenvironment within the pulmonary vasculature.

Regarding pro-inflammatory factors, NF-κB significantly activates the transcription of genes such as TNF-α, IL-1β, IL-6, and IL-8. The mRNAs transcribed from these genes are translated into mature proteins within the cytoplasm and subsequently released into the extracellular space via exocytosis. TNF-α, as a potent pro-inflammatory factor, can bind to TNF receptors on the surface of endothelial cells via an autocrine mechanism, further activating the IKK-NF-κB pathway. The synergistic action of TNF-α and IL-1β enhances the pro-inflammatory phenotype of endothelial cells, promoting the release of additional inflammatory mediators. IL-6, a multifunctional inflammatory cytokine, not only recruits inflammatory cells to migrate locally towards pulmonary vessels but also directly activates proliferation signals in pulmonary vascular smooth muscle cells, laying the groundwork for vascular remodellin (Jiang et al., 2021).

Reduced expression of Claudin-5 and Occludin not only enhances vascular permeability but also disrupts endothelial cell polarity and signalling functions, thereby amplifying contractile and proliferative signals in smooth muscle cells. Endothelial dysfunction further manifests as abnormal expression of pro-inflammatory factors and adhesion molecules, exacerbating inflammatory responses and vascular injury. Hypoxia and ROS activate the NF-κB pathway within endothelial cells, promoting the expression of adhesion molecules such as ICAM-1 and VCAM-1. Once transported to the endothelial cell membrane surface, these molecules bind to ligands like LFA-1 and VLA-4 on inflammatory cell surfaces, mediating the adhesion and infiltration of inflammatory cells. Infiltrating inflammatory cells release substantial quantities of inflammatory mediators, further suppressing eNOS activity and disrupting adherens and tight junctions. This metabolic reprogramming is primarily driven by HIF-1α and the PI3K/Akt pathway, with core mechanisms involving functional regulation of key glycolytic enzymes and the nuclear receptor peroxisome proliferator-activated receptor gamma (PPARγ).

The abnormal activation of the glycolytic pathway constitutes a core feature of metabolic reprogramming in vascular cells within HAPH. Hypoxia significantly enhances the gene expression and protein synthesis of key glycolytic molecules, including Glucose Transporter 1 (GLUT1), Pyruvate Kinase M2 (PKM2), and Lactate Dehydrogenase A (LDHA), through activation of HIF-1α and the PI3K/Akt pathway. As a glucose transporter on the cell membrane, elevated GLUT1 expression enhances cellular glucose uptake capacity, providing ample substrates for cellular metabolism under hypoxic conditions. PKM2, a pivotal kinase in glycolysis, exhibits low catalytic activity and high nuclear translocation capacity. In the cytoplasm, it regulates glycolytic rate to generate modest ATP while producing metabolic intermediates such as pyruvate and lactate (Caruso et al., 2017). Upon nuclear translocation, PKM2 functions as a transcriptional coactivator, binding to transcription factors such as HIF-1α to promote the expression of proliferation-related genes including VEGF and Cyclin D1 (Kim et al., 2006). LDHA catalyses the conversion of pyruvate to lactate, completing the final step of anaerobic glycolysis while maintaining NAD^+^/NADH equilibrium to sustain glycolytic activity. In vascular cells under HAPH, enhanced anaerobic glycolysis leads to substantial accumulation of lactate within the intracellular space and the extracellular gap (Chen J. et al., 2023). Intracellular lactate influences enzyme activity by modulating pH, thereby promoting smooth muscle cell proliferation and contraction (Hu et al., 2022). Extracellular lactate functions as a signalling molecule, binding to G protein-coupled receptors on inflammatory cell surfaces to activate the NF-κB pathway. This facilitates the release of inflammatory mediators, further amplifying inflammatory responses and vascular injury. Concurrently, intermediates from glycolysis serve as precursors for nucleotide and amino acid synthesis, providing material support for smooth muscle cell proliferation and endothelial cell repair (Yuan et al., 2020). PPARγ-mediated lipid metabolism dysregulation represents another critical dimension of metabolic reprogramming. As a member of the nuclear receptor superfamily, PPARγ binds to promoter regions of lipid metabolism-related genes, inhibiting fatty acid synthase expression while promoting fatty acid oxidation to maintain lipid homeostasis. Prolonged hypoxia and inflammatory cytokines significantly suppress PPARγ expression and activity within endothelial and smooth muscle cells. Following reduced PPARγ expression, its inhibitory effect on FASN is lifted, leading to increased fatty acid synthesis and substantial intracellular lipid accumulation. This lipid accumulation alters cell membrane fluidity and signalling functions, promoting a shift in smooth muscle cells from a ‘contractile’ to a ‘proliferative’ state, thereby enhancing cellular proliferation and migration capabilities (Li S. et al., 2024). Concurrently, active lipid mediators generated during lipid metabolism further activate inflammatory signals and contraction pathways, synergistically exacerbating vascular dysfunction. In HAPH-treated smooth muscle cells, PPARγ expression levels negatively correlate with both lipid accumulation and smooth muscle cell proliferative activity. Activation of PPARγ reduces lipid accumulation by inhibiting FASN expression, promotes the transition of smooth muscle cells towards a contractile phenotype, and simultaneously suppresses the PI3K/Akt/mTOR pathway to decrease cell proliferation (Yang, 2025). Furthermore, PPARγ enhances eNOS expression and activity, thereby improving the NO/ET-1 balance and alleviating vasoconstriction, suggesting its role as a key integrator of metabolic, contractile, and inflammatory signalling (Li Y. et al., 2024). Metabolic reprogramming intersects closely with smooth muscle contraction/proliferation and endothelial dysfunction (Li S. et al., 2024). For instance, ATP generated through enhanced glycolysis powers Ca^2+^ pumps and ion channels, sustaining sustained smooth muscle contraction (Yoo and Kim, 2021); lipid accumulation disrupts endothelial tight junctions, increasing vascular permeability; concurrently, the metabolic intermediate lactate activates inflammatory signalling, further inhibiting PPARγ activity and eNOS function.

#### 3.2.3 Hypoxia-induced proliferation of outer membrane fibroblasts

**FIGURE 1:**
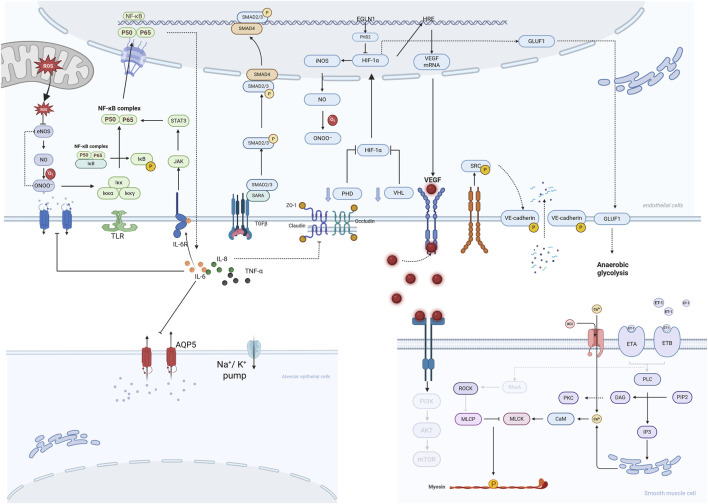
Pathogenesis of HAPE and HAPH.

Prolonged hypoxia coupled with persistent stimulation by inflammatory cytokines leads to damage and even apoptosis of pulmonary vascular endothelial cells. These cells adhere to the endothelial surface and ultimately breach the vascular endothelial barrier, infiltrating the pulmonary interstitium. These infiltrating inflammatory cells undergo further activation locally within pulmonary vessels, releasing substantial quantities of pro-inflammatory factors. These factors, in turn, activate the NF-κB pathway within endothelial cells, promoting the release of additional adhesion molecules and pro-inflammatory factors. This cyclical process transforms the inflammatory response from an initial autonomous activation of endothelial cells into a synergistic activation involving both endothelial and inflammatory cells, significantly amplifying the inflammatory effect. Within the chronic inflammatory microenvironment of HAPH, its primary function shifts towards promoting fibrosis and vascular remodelling. This functional transition is closely associated with the persistent presence of inflammatory mediators. Pro-inflammatory factors bind to corresponding receptors on the surface of smooth muscle cells, activating downstream PI3K/Akt and MAPK/ERK signalling pathways. This promotes the expression of the cell cycle protein Cyclin D1, accelerating cell proliferation. Concurrently, they inhibit smooth muscle cell apoptosis by upregulating the anti-apoptotic protein Bcl-2 and downregulating the pro-apoptotic protein Bax activity, thereby reducing cell death. This leads to an increase in smooth muscle cell numbers and thickening of the pulmonary arteriolar smooth muscle layer (Nör et al., 1999). Concurrently, the sustained activation of NF-κB provides the requisite inflammatory microenvironment for the transcriptional function of Smad2/3. The synergistic interaction between these two pathways perpetuates the fibrotic process. Furthermore, ROS, as an initial signal in the inflammatory response, can also enhance the stability and nuclear translocation capacity of Smad2/3 through oxidative modification, thereby amplifying fibrotic signals (Ma et al., 2025). The transcriptional activation of the TGF-β gene is similarly regulated by HIF-1α. Following TGF-β protein release, it acts via autocrine and paracrine mechanisms on pulmonary vascular endothelial cells and smooth muscle cells, activating the Smad signalling pathway. This promotes the synthesis of extracellular matrix components such as collagen and fibronectin, initiating vascular fibrosis (Wang J. et al., 2025). Even during the initial stages of hypoxia, the early expression of TGF-β can induce mild fibrosis in the pulmonary vascular wall (Ma et al., 2025). GLUT1, LDHA, and PKM2 represent core regulatory targets. Upon activation of HIF-1α, the transcriptional activity of these three genes is significantly enhanced. Their expressed products localise to the cytoplasm of pulmonary vascular endothelial cells and smooth muscle cells, collectively regulating cellular metabolic mode switching (Richalet et al., 2023). As a glucose transporter, elevated GLUT1 expression enhances cellular glucose uptake capacity, supplying ample substrates for cellular metabolism under hypoxic conditions. LDHA catalyses the conversion of pyruvate to lactate, completing the final step of anaerobic glycolysis. PKM2, a key kinase in the glycolytic pathway, regulates glycolytic rate to provide cells with energy and biosynthetic precursors. The pathogenesis of hypoxia-induced HAPE and HAPH is illustrated in Figure 1.

## 4 Chinese herbal medicine prevention and treatment measures

Chinese Herbal Medicine achieves precise therapeutic effects by purifying core active constituents that target key pathological mechanisms of high-altitude hypoxia-induced pulmonary injury, including hypoxic adaptation disorders, inflammatory oxidative stress, vasoconstriction and remodelling, and vascular permeability. It demonstrates significant efficacy in regulating the HIF-1α pathway, suppressing inflammatory responses and oxidative stress damage, and protecting vascular endothelium. Furthermore, its active components are well-defined and dosage administration is controllable.

### 4.1 Single-ingredient Chinese herbal medicines

#### 4.1.1 Ginseng

Ginseng comprises the dried roots and rhizomes of plants belonging to the genus Panax within the Araliaceae family. Its core active constituents are ginsenosides, with ginsenosides Rg1, Rg3, Rb1, and Rb3 demonstrating significant efficacy in preventing and treating pulmonary disorders induced by high-altitude hypoxia. Ginseng extracts are suitable for treating the chronic phase of HAPH and maintaining recovery during the HAPE convalescent phase. The core mechanism of action for ginsenoside Rg1 involves regulating the AMPK/PI3K/AKT signalling pathway, inhibiting proliferation and migration of pulmonary vascular smooth muscle cells, while simultaneously promoting eNOS expression, increasing NO production, and improving vasodilatory function (Li N. et al., 2023; Tian G. et al., 2023). Extensive research indicates that ginsenoside Rg1 reverses pulmonary vascular remodelling in HAPH rats, reduces inflammatory responses, and decreases right ventricular hypertrophy indices (Tang et al., 2023). It ameliorates hypoxia-induced endothelium-dependent vasodilation, elevates NO and eNOS expression, and mitigates oxidative stress, inflammation, and mitochondrial uptake (Zhang et al., 2025). Ginsenoside Rg3 alleviates HAPE by activating the PI3K/AKT pathway to inhibit ferroptosis, stabilising pulmonary vascular endothelial tight junctions, and reducing inflammatory cytokine release and oxidative stress (He et al., 2025). Concurrently, it improves HAPH by suppressing the RhoA/ROCK pathway and calcium overload, thereby decreasing pulmonary arterial smooth muscle contractility (Liu J. et al., 2025). The primary mechanism of ginsenoside Rb1 involves blocking voltage-gated Ca^2+^ channels, reducing Ca^2+^ influx, and alleviating pulmonary vasoconstriction, making it suitable for HAPH patients with concomitant pulmonary vasospasm. Ginsenoside Rb1 diminishes hypoxia-induced pulmonary vasoconstriction while simultaneously inhibiting Ca^2+^-dependent NF-κB pathway activation, thereby reducing inflammatory cytokine release (Wang et al., 2015). Its advantage lies not only in improving pulmonary vascular function but also in regulating the body’s overall qi and blood status, enhancing cardiopulmonary function and hypoxia tolerance. It is particularly suitable for patients presenting with qi deficiency symptoms such as fatigue and lassitude. Ginsenoside Rb3 exerts antioxidant effects by enhancing SOD and CAT activity to reduce ROS levels (Wang et al., 2014), while inhibiting NF-κB to exert anti-inflammatory effects. It suppresses the p38 MAPK pathway, stabilises tight junctions in pulmonary vascular endothelium, reduces vascular permeability, and alleviates pulmonary oedema. Simultaneously inhibiting vascular smooth muscle cell proliferation by blocking calcium channels and the RhoA/ROCK pathway, thereby reducing pulmonary arterial contractility and ameliorating pulmonary arterial hypertension (Sun et al., 2019).

#### 4.1.2 Panax notoginseng

Panax notoginseng refers to the dried roots and rhizomes of plants belonging to the Panax genus within the Araliaceae family. Its core active component is total saponins, which are the principal constituents responsible for its anti-inflammatory, antioxidant, and vasoprotective functions. It exerts therapeutic effects through three mechanisms: firstly, by activating the Nrf2/HO-1 pathway to scavenge ROS and reduce endothelial cell apoptosis; secondly, by downregulating the NF-κB pathway to inhibit IL-6 and TNF-α release, thereby mitigating inflammatory damage; and thirdly, by suppressing pulmonary vascular smooth muscle proliferation, delaying vascular remodelling, promoting alveolar fluid clearance, and alleviating pulmonary oedema (Huang et al., 2024; Pei et al., 2023).

#### 4.1.3 Astragalus

Astragalus refers to the dried root of plants belonging to the Astragalus genus within the Fabaceae family. Its core active constituents include Astragalus polysaccharides (APS) and Astragaloside IV, among others. Astragalus polysaccharides are the primary components responsible for its anti-inflammatory, antioxidant, and endothelial protective functions (Liu H. et al., 2025). Astragalus extracts are indicated for moderate-to-severe cases of HAPE and HAPH. The mechanisms of action for Astragalus polysaccharides primarily include: firstly, activating the Nrf2/HO-1 antioxidant pathway to scavenge ROS and reduce pulmonary vascular endothelial cell apoptosis (Sha et al., 2023); secondly, downregulating the NF-κB pathway to diminish inflammatory cytokine release and mitigate pulmonary tissue inflammatory injury (Ming et al., 2022); thirdly, inhibiting endothelium-mesenchymal transition, reducing collagen deposition in pulmonary vascular walls, and delaying vascular remodelling. Astragaloside IV exhibits a mechanism of action similar to astragalus polysaccharides, with more pronounced effects in inhibiting pulmonary vascular smooth muscle proliferation. It reduces the proliferation rate of pulmonary vascular smooth muscle cells in HAPH model rats while promoting alveolar fluid clearance and improving pulmonary oedema (Jin et al., 2021).

#### 4.1.4 Rhodiola

Rhodiola is a classic Chinese medicinal herb for preventing and treating high-altitude sickness. It comprises the dried roots and rhizomes of plants belonging to the Rhodiola genus within the Crassulaceae family. Its core active constituents include salidroside, tyrosol, and quercetin (Qu et al., 2020), with salidroside being the primary component responsible for its anti-hypoxic and anti-inflammatory effects (Yang et al., 2023). Rhodiola extracts are suitable for the prevention and mild treatment of HAPE and HAPH, with their mechanism of action primarily manifested in four aspects: firstly, inhibiting the excessive activation of the HIF-1α pathway to reduce the release of downstream pro-angiogenic molecules such as VEGF and ET-1, thereby suppressing pulmonary vasoconstriction and vascular leakage (Cao et al., 2022); Secondly, they enhance antioxidant enzyme activity, scavenge reactive oxygen species (ROS), mitigate lipid peroxidation damage, and protect pulmonary vascular endothelial function (Hou et al., 2023; Li et al., 2021). Thirdly, they modulate inflammatory factor balance by downregulating pro-inflammatory factor expression and upregulating anti-inflammatory factor levels, thereby alleviating pulmonary tissue inflammatory responses (Wang, 2023). Fourthly, they inhibit pulmonary vascular proliferation and angiogenesis by suppressing the PI3K/Akt/mTOR pathway (Yang et al., 2025). Basic experiments confirm that rhodiolol can inhibit hypoxia-induced increases in pulmonary arterial pressure while simultaneously reducing vascular permeability and pulmonary vascular fluid extravasation, thereby decreasing pulmonary water content in HAPE model rats (Huang et al., 2022; Wang, 2023). The core mechanism of quercetin involves inhibiting the NF-κB/STAT3 pathway to reduce inflammatory cytokine release, while simultaneously regulating the PI3K/AKT/mTOR pathway to suppress pulmonary vascular smooth muscle proliferation. Studies indicate that quercetin nanoliposomes reduce pulmonary artery pressure by 28% in HAPH rats, decrease vascular wall thickness by 23% (P < 0.01), and lower inflammatory cytokine TNF-α levels by 5% (P < 0.01). Basic experiments confirmed that quercetin enhances antioxidant enzyme activities such as SOD and GSH-Px, reduces malondialdehyde (MDA) production, protects pulmonary vascular endothelial function, and simultaneously inhibits VEGF expression, thereby reducing angiogenesis and vascular remodelling (Lin et al., 2025; Tripathi et al., 2021).

#### 4.1.5 Eleutherococcus senticosus

Eleutherococcus senticosus comprises the dried roots and rhizomes of plants belonging to the genus Eleutherococcus within the Araliaceae family. Its core active constituents are eleutheroside B and eleutheroside E, which are primarily responsible for its anti-inflammatory, antioxidant, and pulmonary vasoprotective effects. Its mechanisms include: firstly, activating the Nrf2/HO-1 pathway to scavenge ROS and reduce endothelial cell apoptosis; secondly, activating the AMPK/mTOR signalling pathway to restore impaired autophagy flux; and thirdly, exerting anti-inflammatory effects by inhibiting inflammasomes (Pei et al., 2024; Shen et al., 2023; Wang et al., 2022). The relevant mechanisms of single Chinese herbal medicines in preventing and treating high-altitude hypoxia-induced pulmonary diseases are summarised in Table 1.

### 4.2 Compound preparations of traditional Chinese medicine

Compound preparations of traditional Chinese medicine, grounded in the theory of syndrome differentiation and treatment, achieve synergistic effects through the combination of multiple herbs. This approach addresses both symptomatic relief and underlying mechanisms, making them suitable for disease prevention, the management of mild cases, and the treatment of moderate to severe conditions. These formulations demonstrate well-established clinical efficacy and a high safety profile.

#### 4.2.1 Danshen-Astragalus Extract Granules

Danshen-Astragalus Extract Granules comprise a combination of four traditional Chinese medicinal herbs: Astragalus membranaceus, Salvia miltiorrhiza, Polygonatum sibiricum, and Ligusticum chuanxiong. Its core active constituents are Astragalus polysaccharides, Tanshinone IIA, Tanshinone, Polygonatum polysaccharides, and Ligustrazine. Its mechanism of action is as follows: Astragalus polysaccharides synergistically inhibit the TGF-β1/Smad pathway with Danshinkon IIA to reduce collagen deposition; Danshenin suppresses hypoxia-induced proliferation of pulmonary arterial smooth muscle cells, thereby alleviating hypoxic pulmonary arterial hypertension in rats (Zhang et al., 2018); Polygonatum polysaccharides enhance antioxidant capacity and improve mitochondrial function (Yu and Yu, 2024); and chuanxiongzine improves microcirculation and promotes pulmonary vascular repair (Shi, 2003). Danshen-Astragalus Granules reduce scores on the Traditional Chinese Medicine Syndrome Grading and Quantification Scale, ameliorating clinical symptoms in individuals experiencing acute high-altitude sickness. They may exert protective effects on those acutely exposed to high altitudes by lowering serum TNF-α and IL-6 levels (Li M. et al., 2023).

#### 4.2.2 Compound Danshen Drops

Compound Danshen Drops comprise a combination of three traditional Chinese medicinal ingredients: Salvia miltiorrhiza, Panax notoginseng, and borneol. Its core active constituents are tanshinone IIA, ginsenoside Rg1, and ginsenoside Rb1. This formulation is indicated for the adjunctive treatment of both haemorrhagic and non-haemorrhagic pulmonary oedema, proving particularly suitable for patients presenting with concomitant pulmonary circulatory impairment and elevated blood viscosity. These components synergistically improve pulmonary circulation, reduce thrombus formation, and protect vascular endothelium. Their mechanisms include: Danshen-tong IIA promotes nitric oxide release, enhancing pulmonary vasodilation (Zhang et al., 2022); Panax notoginsenosides inhibit platelet aggregation and lower blood viscosity (Huang et al., 2023); Borneol enhances drug bioavailability, facilitating distribution of active components within pulmonary tissue. This formulation has been approved by the National Medical Products Administration for adjunctive treatment of high-altitude sickness and is included as a recommended medication in the Guidelines for Diagnosis and Treatment of High-Altitude Sickness (2022 Edition).

#### 4.2.3 Compound rhodiola rhabdoides oral liquid

Compound Rhodiola Rhabdoides Oral Liquid is formulated from three Chinese medicinal herbs: Rhodiola rosea, Astragalus membranaceus, and Lycium barbarum. Its core active constituents comprise rhodiolansides, astragalus polysaccharides, and lycium polysaccharides. These three ingredients synergistically enhance the body’s tolerance to hypoxia and reduce vascular leakage. Their mechanisms of action are as follows: Rhodiola glycosides inhibit the HIF-1α pathway, reducing the release of ET-1 and VEGF (Zhou et al., 2021); Astragalus polysaccharides activate the Nrf2/HO-1 pathway, enhancing antioxidant capacity (Sha et al., 2023); Lycium polysaccharides modulate immune function, reducing the release of inflammatory cytokines (Xiang et al., 2025).

Beyond these classical formulations, several novel Chinese herbal combinations have demonstrated promising efficacy in managing high-altitude pulmonary diseases caused by hypoxia. For instance, Ginseng-Ophiopogon Injection (containing ginseng and ophiopogon) features core active components ginsenosides and ophiopogon polysaccharides, which improve myocardial metabolism and protect pulmonary vascular endothelium (Zheng et al., 2012). Modified Minor Blue Dragon Decoction (Ephedra, Cinnamomum cassia, Asarum, Zingiber officinale, etc.) is indicated for HAPE patients. This formula alleviates pulmonary inflammation and ameliorates high-altitude pulmonary oedema, acting through regulation of PI3K/Akt/mTOR and HIF-1α pathway expression (Wang R. R. et al., 2025). Yunyao Qilongtian (Panax notoginseng, Rhodiola rosea, Lumbricus terrestris, etc.) (Fu et al., 2025), with core active components ginsenosides and rhodiolosides, inhibits angiogenesis and pulmonary arteriolar remodelling by reducing VEGF protein expression in hypoxic lung tissue (Cao et al., 2022; Chen, 2023). These compound formulations are all based on the principles of pattern differentiation and treatment in traditional Chinese medicine, precisely combining ingredients according to different syndromes, reflecting the advantages of individualised treatment with Chinese herbal medicine. The mechanisms of Chinese herbal compound formulations in preventing and treating pulmonary diseases caused by high-altitude hypoxia are shown in Table 2.

## 5 Conclusion and prospects

The pathogenesis of high-altitude hypoxia-induced pulmonary diseases can be summarised as hypoxia perception-inflammation activation-vascular injury centred on hypoxia. Hypoxia inhibits PHD activity, leading to the stable accumulation and nuclear translocation of HIF-1α. This activates the transcription of target genes such as VEGF and ET-1, constituting the initial signal for disease onset. ROS generated by mitochondrial electron transport chain dysfunction further activate the NF-κB pathway, releasing pro-inflammatory factors and adhesion molecules that exacerbate inflammatory infiltration in lung tissue. Ultimately, this disrupts vascular endothelial junction proteins such as VE-cadherin and claudins, increasing vascular permeability. Concurrently, abnormalities in ET-1-mediated calcium signalling pathways and potassium channel function induce pulmonary vasoconstriction and abnormal smooth muscle cell proliferation, resulting in the acute pulmonary oedema of HAPE and the chronic vascular remodelling of HAPH. The cross-regulation between HIF-1α and NF-κB pathways, NO/ET-1 imbalance, and metabolic reprogramming constitute pivotal mechanisms sustaining this pathological network.

Chinese Herbal Medicine extracts focus on precisely targeting pathological pathways through identified active components. Compound formulations, grounded in CHM syndrome differentiation and treatment principles, achieve synergistic effects through multiple components, particularly addressing limitations in long-term maintenance and recurrence reduction where conventional interventions fall short. Future research requires a more systematic and in-depth approach to elucidate the holistic therapeutic mechanisms of CHM’s multi-component, multi-targeted action. Although CHM demonstrates significant potential in preventing and treating pulmonary diseases caused by high-altitude hypoxia, subsequent large-scale, multicentre clinical trials of traditional Chinese medicine formulations should be conducted using standardised efficacy evaluation metrics. Leveraging network pharmacology, molecular biology, and metabolomics technologies, the interaction targets between traditional Chinese medicine’s multiple components and pathological networks should be analysed. This will elucidate the regulatory relationships between components, targets, and pathways, exploring the synergistic regulatory mechanisms of CHM. This will advance formulation optimisation and quality control, enabling the development of novel lung-targeted, long-acting, portable dosage forms that enhance CHM bioavailability and clinical applicability. Furthermore, establishing quality control standards based on fingerprint profiling and quantitative analysis will ensure the consistent quality of CHM extracts and compound formulations. When using CHM to treat lung conditions caused by high-altitude hypoxia, it should be combined with other Traditional Chinese Medical therapies such as a acupuncture and moxibustion. As a distinctive non-pharmacological therapy within CHM, acupuncture and moxibustion regulates the flow of qi and blood through the meridians and improves organ function. When used in conjunction with herbal medicine, it further enhances the prevention and treatment of hypoxic lung injury at high altitudes, better utilising the holistic regulatory benefits of Traditional Chinese Medicine and providing a more comprehensive approach to the clinical management of such conditions.

The pathogenesis of high-altitude pulmonary injury is primarily driven by a hypoxia-induced cascade involving oxidative stress, inflammatory responses, and vascular endothelial dysfunction. CHM offers significant therapeutic advantages by exerting multi-target and synergistic effects against these interconnected pathways. Future efforts should focus on deepening the integration of basic research and clinical translation to refine evidence-based practices, enhance mechanistic understanding, and optimise formulation quality. Collectively, these efforts will advance the standardized and precise application of CHM, thereby strengthening health protection for populations exposed to high-altitude environment.
