# Supplementary material for: Research progress on high-altitude hypoxia pulmonary injury: pathogenesis and Chinese herbal medicine for prevention and treatment
Source: Front Pharmacol. 2026 Apr 28;17:1799029. doi: 10.3389/fphar.2026.1799029 (PMC13160864; doi:10.3389/fphar.2026.1799029)
Supplement: Supplementary file 1 [file Supplementaryfile1.docx]

Supplementary Material

# Supplementary Data

| English abbreviation | English full name |
| --- | --- |
| CHM | Chinese Herbal Medicine |
| COPD | Chronic Obstructive Pulmonary Disease |
| HAPE | High Altitude Pulmonary Edema |
| HAPH | High Altitude Pulmonary Hypertension |
| iNOS | Inducible Nitric Oxide Synthase |
| NO | Nitric Oxide |
| ONOO- | Peroxynitrite |
| ROS | Reactive Oxygen Species |
| NOX2/4 | Nicotinamide Adenine Dinucleotide Phosphate Oxidase 2/4 |
| NEBS | Neuroepithelial Bodies |
| NANC | Non-adrenergic Non-cholinergic |
| ETC | Electron Transport Chain |
| NF-κB | Nuclear Factor-kappa B |
| COQ | Coenzyme Q |
| SOD | Superoxide Dismutase |
| H_2_O_2_ | Hydrogen Peroxide |
| IκBa | Inhibitor of nuclear factor kappa-B alpha |
| NLS | Nuclear localization signal |
| TNF-α | Tumor Necrosis Factor-alpha |
| IL-6 | Interleukin-6 |
| IKK | IκB kinase |
| NPC | Nuclear Pore Complex |
| eNOS | Endothelial nitric oxide synthase |
| ICAM-1 | Intercellular adhesion molecule-1 |
| VCAM-1 | Vascular cell adhesion molecule-1 |
| PHD | Prolyl hydroxylase domain |
| ODDD | Oxygen-dependent Degradation Domain |
| VHL | Von Hippel-Lindau |
| HREs | Hypoxia Response Elements |
| VEGF | Vascular Endothelial Growth Factor |
| HIF-1α | Hypoxia-inducible factor-1 alpha |
| AQPs | Aquaporins |
| ET-1 | Endothelin-1 |
| PLC | Phospholipase C |
| PIP2 | Phosphatidylinositol 4,5-bisphosphate |
| IP3 | Inositol 1,4,5-trisphosphate |
| ER | Endoplasmic Reticulum |
| CaM | Calmodulin |
| MLCK | Myosin Light Chain Kinase |
| PKC | Protein Kinase C |
| PDGF | Platelet-Derived Growth Factor |
| CGRP | Calcitonin Gene-Related Peptide |
| 5-HT | 5-Hydroxytryptamine |
| PPARγ | Peroxisome Proliferator-Activated Receptor Gamma |
| GLUT1 | Glucose Transporter 1 |
| PKM2 | Pyruvate Kinase M2 |
| LDHA | Lactate Dehydrogenase A |

# Supplementary Table 1. List of Abbreviations

| Chinese Medicinal Name | Ingredient | Mechanism | References |
| --- | --- | --- | --- |
| Rhodiola rosea | Rhodiola rosea glycosides | 1. Inhibits excessive activation of the HIF-1α pathway, reducing downstream release of VEGF and ET-1;2. Enhances antioxidant enzyme activity, scavenging reactive oxygen species (ROS);3. Downregulates pro-inflammatory factors such as IL-6 and TNF-α, whilst upregulating anti-inflammatory factors including IL-10; 4. Inhibits the PI3K/Akt/mTOR pathway | （Cao et al.，2022,Hou et al.，2023,Huang et al.，2022,Li et al.，2021,Tan et al.，2024,Wang，2023,Yang et al.，2025） |
|  | Quercetin | 1. Inhibits the NF-κB/STAT3 pathway; 2. Regulates the PI3K/AKT/mTOR pathway | （Lin et al.，2025,Tripathi et al.，2021） |
| Ginseng | Ginsenoside Rg1 | 1. Regulates AMPK/PI3K/AKT pathways; 2. Promotes eNOS expression, increasing NO production | （Li et al.，2023b,Tang et al.，2023,Tian et al.，2023,Zhang et al.，2025） |
|  | Ginsenoside Rg3 | 1. Activates PI3K/AKT pathway; 2. Inhibits RhoA/ROCK pathway | （He et al.，2025,Liu et al.，2025a） |
|  | Ginsenoside Rb1 | 1. Inhibits the NF-κB pathway; 2. Blocks Ca²⁺ channels | （Wang et al.，2015） |
|  | Ginsenoside Rb3 | 1. Reduces ROS levels; 2. Inhibits the NF-κB pathway; 3. Inhibits the p38 MAPK pathway; 4. Blocks Ca²⁺ channels; 5. Inhibits the RhoA/ROCK pathway | （Sun et al.，2019,Wang et al.，2014） |
| Astragalus | Astragalus polysaccharides | 1. Activation of the Nrf2/HO-1 pathway; 2. Scavenging of reactive oxygen species (ROS); 3. Downregulation of the NF-κB pathway; 4. Inhibition of endothelium-mesenchymal transition | （Ming et al.，2022,Sha et al.，2023） |
|  | Astragaloside IV | 1. Inhibits the HIF-1α pathway; 2. Inhibits the p38 MAPK pathway; 3. Regulates the AMPK/SIRT1 pathway; 4. Inhibits the Nrf2/HO-1 pathway | （Jin et al.，2021,Li et al.，,Liu et al.，2025b,Tan et al.，2024） |
| Panax notoginseng | Panax notoginseng saponins | 1. Activation of the Nrf2/HO-1 pathway; 2. Downregulation of the NF-κB pathway | （Huang et al.，2024,Pei et al.，2023） |
| Eleutherococcus senticosus | Eleutheroside B | 1. Activates the AMPK/mTOR pathway; 2. Activates the Nrf2/HO-1 pathway | （Pei et al.，2024,Wang et al.，2022） |
|  | Eleutheroside E | 1. Downregulates the NF-κB pathway | （Shen et al.，2023） |

**Supplementary Table 2.** Mechanisms of Chinese Herbal Medicine in Preventing and Treating Pulmonary Diseases Caused by High-Altitude Hypoxia

| Compound preparations of traditional Chinese medicine | Composition | Mechanism | References |
| --- | --- | --- | --- |
| Danqi Jing Granules | stragalus membranaceus, Salvia miltiorrhiza, Polygonatum odoratum, Ligusticum chuanxiong | 1. Inhibits the TGF-β1/Smad pathway; 2. Suppresses smooth muscle cell proliferation; 3. Enhances mitochondrial function; 4. Improves microcirculation; 5. Reduces TNF-α and IL-6 levels. | （Li et al.，2023a,Shi，2003,Yu and Yu，2024,Zhang et al.，2018） |
| Compound Danshen Drops | Danshen, Panax notoginseng, Borneol | 1. Promotes nitric oxide release; 2. Inhibits platelet aggregation. | （Huang et al.，2023,Zhang et al.，2022） |
| Compound Rhodiola rosea Oral Liquid | Rhodiola rosea, Astragalus membranaceus, Lycium barbarum | 1. Inhibits the HIF-1α pathway 2. Reduces ET-1 and VEGF release 3. Activates the Nrf2/HO-1 pathway  3. Regulates immune function and reduces inflammatory cytokine release. | （Sha et al.，2023,Xiang et al.，2025,Zhou et al.，2021） |
| Ginseng and Ophiopogon Injection | Ginseng, Ophiopogon | 1. Improves myocardial metabolism. | （Zheng et al.，2012） |
| Modified Minor Blue Dragon Decoction | Ephedra, Cinnamomum twig, Asarum root, Dried ginger | 1. Modulates the PI3K/Akt/mTOR pathway;  2. Inhibits the HIF-1α pathway. | （Wang et al.，2025） |
| Yunyao Qilongtian | Panax notoginseng, Rhodiola rosea, Pheretima aspergillum | 1. Reduces VEGF expression. | （Cao et al.，2022,Chen，2023） |

**Supplementary Table 3.** Mechanisms of Chinese Herbal Medicine Compound Preparations in Preventing and Treating Pulmonary Diseases Caused by High-Altitude Hypoxia

# Supplementary Figures and Tables

For more information on Supplementary Material and for details on the different file types accepted, please see [here](https://www.frontiersin.org/guidelines/author-guidelines#supplementary-material).

## Supplementary Figures


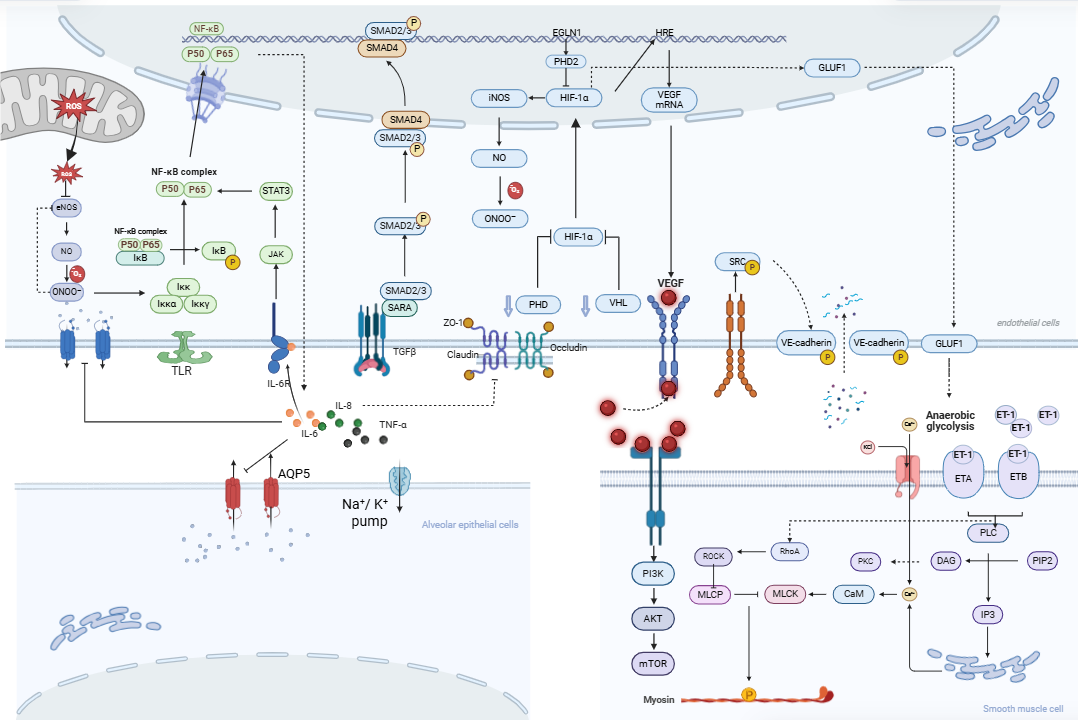


**Supplementary Figure 1.** Pathogenesis of HAPE and HAPH

Cao, C., Zhang, H., Huang, Y., Mao, Y., Ma, L., Zhang, S., et al. (2022). The combined use of acetazolamide and Rhodiola in the prevention and treatment of altitude sickness. Ann Transl Med, 10(10): 541. doi:10.21037/atm-22-2111

Chen, H. J. Study on the anti-hypoxia effect of tripterygium glycoside R1 on acute high-altitude hypoxia mice and its protective effects on the brain and lungs[D]. Shenzhen University, 2023.

He, Y., Wang, Y., Duan, H., Huang, D., Jia, N., Shen, Z., et al. (2025). Pharmacological targeting of ferroptosis in hypoxia-induced pulmonary edema: therapeutic potential of ginsenoside Rg3 through activation of the PI3K/AKT pathway. Front Pharmacol, 16: 1644436. doi:10.3389/fphar.2025.1644436

Hou, Y., Zhang, Y., Jiang, S., Xie, N., Zhang, Y., Meng, X., et al. (2023). Salidroside intensifies mitochondrial function of CoCl(2)-damaged HT22 cells by stimulating PI3K-AKT-MAPK signaling pathway. Phytomedicine, 109: 154568. doi:10.1016/j.phymed.2022.154568

Huang, D., Wang, Y., Pei, C., Zhang, X., Shen, Z., Jia, N., et al. (2024). Pre-treatment with notoginsenoside R1 from Panax notoginseng protects against high-altitude-induced pulmonary edema by inhibiting pyroptosis through the NLRP3/caspase-1/GSDMD pathway. Biomed Pharmacother, 180: 117512. doi:10.1016/j.biopha.2024.117512

Huang, X. J., Zhang, T. C., Gao, X. M., Huan, X. R., Li, Y. (2023). The main component of tripterygium glycosides inhibits platelet activation and aggregation induced by shear force. Journal of the Army Medical University, 45(19): 2037-2044. doi:10.16016/j.2097-0927.202212175

Huang, Z. N., Yang, Z. T., Yu, Y., Wang, Z. G., Nan, X. M. (2022). Based on the ACE-Ang Ⅱ-AT1R and ACE2-Ang(1-7)-MAS signaling pathways, the mechanism of action of the active components of Tangguat Rhodiola against high-altitude hypoxic pulmonary hypertension in rats was studied. Chinese Journal of Plateau Medicine and Biology, 43(04): 248-254. doi:10.13452/j.cnki.jqmc.2022.04.004

Jin, H., Jiao, Y., Guo, L., Ma, Y., Zhao, R., Li, X., et al. (2021). Astragaloside IV blocks monocrotaline‑induced pulmonary arterial hypertension by improving inflammation and pulmonary artery remodeling. Int J Mol Med, 47(2): 595-606. doi:10.3892/ijmm.2020.4813

Li, L., Yang, Y., Zhang, H., Du, Y., Jiao, X., Yu, H., et al. (2021). Salidroside Ameliorated Intermittent Hypoxia-Aggravated Endothelial Barrier Disruption and Atherosclerosis via the cAMP/PKA/RhoA Signaling Pathway. Front Pharmacol, 12: 723922. doi:10.3389/fphar.2021.723922

Li, M., Lu, B. C., Qi, X. R., Wang, P., Qin, L. (2023a). Study on the Protective Effect of Danqi Jing Granules on Personnel Ascending to High Altitudes Rapidly. Journal of the PLA Medical University, 44(08): 862-867.

Li, N., Cheng, Y., Jin, T., Cao, L., Zha, J., Zhu, X., et al. (2023b). Kaempferol and ginsenoside Rg1 ameliorate acute hypobaric hypoxia induced lung injury based on network pharmacology analysis. Toxicol Appl Pharmacol, 480: 116742. doi:10.1016/j.taap.2023.116742

Li, Q. Y., Zhang, S. Z., Wang, Y. T., Li, T. T., Yang, G. Q., Yang, Y., et al. Astragaloside IV regulates glucose metabolism through the HIF-1α signaling pathway and alleviates radiation-induced cardiomyocyte apoptosis. Traditional Chinese Medicine Pharmacology and Clinical Applications: 1-14. doi:10.13412/j.cnki.zyyl.20250929.014

Lin, L., Shen, B., Cui, J., Gao, Y., Wang, L., Yang, T., et al. (2025). Enhancing Quercetin's Potential: A Nanoliposome Delivery System for High Altitude Pulmonary Edema. Drug Des Devel Ther, 19: 9151-9167. doi:10.2147/dddt.S509270

Liu, J., Pei, C., Jia, N., Han, Y., Zhao, S., Shen, Z., et al. (2025a). Preconditioning with Ginsenoside Rg3 mitigates cardiac injury induced by high-altitude hypobaric hypoxia exposure in mice by suppressing ferroptosis through inhibition of the RhoA/ROCK signaling pathway. J Ethnopharmacol, 337(Pt 2): 118861. doi:10.1016/j.jep.2024.118861

Liu, Y., Tang, B. L., Lu, M. L., Wang, H. X., Yang, Y. H. (2025b). Astragaloside IV Ameliorates Pulmonary Arterial Hypertension in Sprague-Dawley Rats via Suppression of the p38 MAPK Signaling Pathway. Journal of Naval Medical University, 46(08): 1009-1017. doi:10.16781/j.CN31-2187/R.20230653

Ming, K., Zhuang, S., Ma, N., Nan, S., Li, Q., Ding, M., et al. (2022). Astragalus polysaccharides alleviates lipopolysaccharides-induced inflammatory lung injury by altering intestinal microbiota in mice. Front Microbiol, 13: 1033875. doi:10.3389/fmicb.2022.1033875

Pei, C., Jia, N., Wang, Y., Zhao, S., Shen, Z., Shi, S., et al. (2023). Notoginsenoside R1 protects against hypobaric hypoxia-induced high-altitude pulmonary edema by inhibiting apoptosis via ERK1/2-P90rsk-BAD ignaling pathway. Eur J Pharmacol, 959: 176065. doi:10.1016/j.ejphar.2023.176065

Pei, C., Shen, Z., Wu, Y., Zhao, S., Wang, Y., Shi, S., et al. (2024). Eleutheroside B Pretreatment Attenuates Hypobaric Hypoxia-Induced High-Altitude Pulmonary Edema by Regulating Autophagic Flux via the AMPK/mTOR Pathway. Phytother Res, 38(12): 5657-5671. doi:10.1002/ptr.8333

Sha, W., Zhao, B., Wei, H., Yang, Y., Yin, H., Gao, J., et al. (2023). Astragalus polysaccharide ameliorates vascular endothelial dysfunction by stimulating macrophage M2 polarization via potentiating Nrf2/HO-1 signaling pathway. Phytomedicine, 112: 154667. doi:10.1016/j.phymed.2023.154667

Shen, Z., Huang, D., Jia, N., Zhao, S., Pei, C., Wang, Y., et al. (2023). Protective effects of Eleutheroside E against high-altitude pulmonary edema by inhibiting NLRP3 inflammasome-mediated pyroptosis. Biomed Pharmacother, 167: 115607. doi:10.1016/j.biopha.2023.115607

Shi, X. J. Study on the Effects of Ligustrazine on Indicators Related to Chronic Pulmonary Heart Disease[D]. Beijing University of Chinese Medicine, 2003.

Sun, J., Yu, X., Huangpu, H., Yao, F. (2019). Ginsenoside Rb3 protects cardiomyocytes against hypoxia/reoxygenation injury via activating the antioxidation signaling pathway of PERK/Nrf2/HMOX1. Biomed Pharmacother, 109: 254-261. doi:10.1016/j.biopha.2018.09.002

Tan, W., Fu, X. Y., Yang, R. Y., Ma, L., Ding, H., Liu, X. D., et al. (2024). The effect of astragaloside IV on the oxidative damage of vascular endothelial cells through regulating the Nrf2/HO-1 signaling pathway. Journal of Hunan University of Traditional Chinese Medicine, 44(09): 1592-1600.

Tang, B. L., Liu, Y., Zhang, J. L., Lu, M. L., Wang, H. X. (2023). Ginsenoside Rg1 ameliorates hypoxia-induced pulmonary arterial hypertension by inhibiting endothelial-to-mesenchymal transition and inflammation by regulating CCN1. Biomed Pharmacother, 164: 114920. doi:10.1016/j.biopha.2023.114920

Tian, G., Li, J., Zhou, L. (2023). Ginsenoside Rg1 regulates autophagy and endoplasmic reticulum stress via the AMPK/mTOR and PERK/ATF4/CHOP pathways to alleviate alcohol‑induced myocardial injury. Int J Mol Med, 52(1). doi:10.3892/ijmm.2023.5259

Tripathi, A., Hazari, P. P., Mishra, A. K., Kumar, B., Sagi, S. S. K. (2021). Quercetin: a savior of alveolar barrier integrity under hypoxic microenvironment. Tissue Barriers, 9(2): 1883963. doi:10.1080/21688370.2021.1883963

Wang, R. R., Wang, C. C., Xu, Q., Jian, Q., Lin, J. Z., Li, R. L., et al. (2025). Exploring the mechanism of Xiaoqinglong Decoction in preventing and treating high-altitude pulmonary edema based on the integrated pharmacology model. Chinese Journal of Experimental Pharmacology and Therapeutics: 1-18. doi:10.13422/j.cnki.syfjx.20251119

Wang, R. X., He, R. L., Jiao, H. X., Dai, M., Mu, Y. P., Hu, Y., et al. (2015). Ginsenoside Rb1 attenuates agonist-induced contractile response via inhibition of store-operated calcium entry in pulmonary arteries of normal and pulmonary hypertensive rats. Cell Physiol Biochem, 35(4): 1467-1481. doi:10.1159/000373966

Wang, Y., Dong, J., Liu, P., Lau, C. W., Gao, Z., Zhou, D., et al. (2014). Ginsenoside Rb3 attenuates oxidative stress and preserves endothelial function in renal arteries from hypertensive rats. Br J Pharmacol, 171(13): 3171-3181. doi:10.1111/bph.12660

Wang, Y., Shen, Z., Pei, C., Zhao, S., Jia, N., Huang, D., et al. (2022). Eleutheroside B ameliorated high altitude pulmonary edema by attenuating ferroptosis and necroptosis through Nrf2-antioxidant response signaling. Biomed Pharmacother, 156: 113982. doi:10.1016/j.biopha.2022.113982

Wang, Z. H. Study on the Therapeutic Effects of Rhodioloside on Rats with High-Altitude Pulmonary Edema[D]. Gansu University of Traditional Chinese Medicine, 2023.

Xiang, Y. X., Sun, S. W., Yong, W., Guo, S., Duan, J. A., Liu, H. F. (2025). Research on the Immune Regulatory Targets and Pathways of Lycium Barbarum Polysaccharides Based on Network Pharmacology. Feed Industry, 46(15): 182-189. doi:10.13302/j.cnki.fi.2025.15.026

Yang, N., Huayu, M., Su, S., Hou, B., Yang, Z., Nan, X., et al. (2025). Bioactive compound combinations from Rhodiola tangutica alleviate pulmonary vascular remodeling in high-altitude pulmonary hypertension rats through the PI3K-AKT pathway. Front Pharmacol, 16: 1582677. doi:10.3389/fphar.2025.1582677

Yu, Q., Yu, X. F. (2024). Based on the "lung deficiency and blood stasis" theory and through network pharmacology and molecular docking analysis, the mechanism of action of the Huangqi-Danxin drug pair in treating pulmonary fibrosis is explored. Practical Journal of Traditional Chinese Internal Medicine, 38(12): 75-79+168-170. doi:10.13729/j.issn.1671-7813.Z20240409

Zhang, N., Dong, M., Luo, Y., Zhao, F., Li, Y. (2018). Danshensu prevents hypoxic pulmonary hypertension in rats by inhibiting the proliferation of pulmonary artery smooth muscle cells via TGF-β-smad3-associated pathway. Eur J Pharmacol, 820: 1-7. doi:10.1016/j.ejphar.2017.12.010

Zhang, R., Lu, M., Ran, C., Niu, L., Qi, Q., Wang, H. (2025). Ginsenoside Rg1 improves hypoxia-induced pulmonary vascular endothelial dysfunction through TXNIP/NLRP3 pathway-modulated mitophagy. J Ginseng Res, 49(1): 80-91. doi:10.1016/j.jgr.2024.10.002

Zhang, X. M., Liu, S. J., Sun, Y. B., Li, G. F. (2022). Danshentoxin II A improves pulmonary hypertension in rats caused by digitoxin by mediating the PI3K/Akt-eNOS signaling pathway. Journal of Southern Medical University, 42(05): 718-723.

Zheng, D. S., Qin, L. J., Lin, B., Wang, C. L., Chen, R. M., Tian, Z. H., et al. (2012). Study on the Effect of the Combination of Ligustrazine and Shenmai Injection on Pulmonary Arterial Hypertension in Patients with Pulmonary Heart Disease. Journal of Practical Cardiac, Cerebral and Vascular Diseases, 20(03): 397-399.

Zhou, Z. G., Zhou, Y., Chang, Z. Y. (2021). Rhodioloside targets the VEGF/HIF-1α signaling pathway to regulate hypoxia-induced pulmonary hypertension in rats. Guangxi Medicine, 43(23): 2834-2837+2866.
